# Leveraging meta-regression to test if medication effects on cue-induced craving are associated with clinical efficacy

**DOI:** 10.1007/s00213-024-06589-7

**Published:** 2024-04-13

**Authors:** Steven J. Nieto, Han Du, Lindsay R. Meredith, Suzanna Donato, Molly Magill, Lara A. Ray

**Affiliations:** 1 Department of Psychology, University of California at Los Angeles, 1285 Franz Hall, Box 951563, Los Angeles, CA 90095-1563 USA; 2 Center for Alcohol and Addiction Studies, Brown University School of Public Health, Providence, Rhode Island USA; 3 Department of Psychiatry and Biobehavioral Sciences, University of California at Los Angeles, Los Angeles, CA USA

**Keywords:** Alcohol cue-reactivity, Alcohol use disorder, Cue-induced craving, Human laboratory, Medication development, Randomized clinical trials

## Abstract

**Rationale:**

The alcohol cue exposure paradigm is a common method for evaluating new treatments for alcohol use disorder (AUD); however, it is unclear if medication-related reductions in cue-induced craving in the human laboratory can predict the clinical success of those medications in reducing alcohol consumption during clinical trials.

**Objectives:**

To use a novel meta-analytic approach to test whether medication effect sizes on cue-induced alcohol craving are associated with clinical efficacy in clinical trials.

**Method:**

We searched the literature for medications tested for AUD treatment using both the alcohol cue-reactivity paradigm and randomized clinical trials (RCTs). For alcohol cue-reactivity studies, we computed medication effect sizes for cue-induced alcohol craving (k = 36 studies, 15 medications). For RCTs, we calculated medication effect sizes for heavy drinking and abstinence (k = 139 studies, 19 medications). Using medication as the unit of analysis, we applied the Williamson-York bivariate weighted least squares estimation to account for errors in both independent and dependent variables. We also conducted leave-one-out cross validation simulations to examine the predictive utility of cue-craving medication effect sizes on RCT heavy drinking and abstinence endpoints.

**Results:**

There was no significant relationship between medication effects on cue-induced alcohol craving in the human laboratory and medication effects on heavy drinking ($\widehat{\beta }$ = 0.253, SE = 0.189, *p* = 0.090) and abstinence ($\widehat{\beta }$ = 0.829, SE = 0.747, *p* = 0.133) in RCTs.

**Conclusions:**

The preliminary results of the current study challenge the assumption that alcohol cue-reactivity alone can be used as an early efficacy indicator for AUD pharmacotherapy development. These findings suggest that a wider range of early efficacy indicators and experimental paradigms be considered for Phase II testing of novel compounds.

**Supplementary Information:**

The online version contains supplementary material available at 10.1007/s00213-024-06589-7.

## Introduction

Developing medications for alcohol use disorder (AUD) is an expensive and lengthy endeavor. In the last twenty years, despite substantial investment, there have been no new AUD medications approved by the Food and Drug Administration (FDA) (Heilig et al. 2016). The standard development process for new AUD medications involves animal testing, human safety testing, efficacy testing in randomized clinical trials (RCTs), and possibly FDA approval (Litten et al. 2012, 2014). Scientists must make critical "go/no-go" decisions at each stage regarding the potential pharmacotherapies. Experimental psychopharmacology paradigms could aid in identifying the early clinical efficacy of compounds in development (Litten et al. 2020). These paradigms allow for the initial detection of medication advantages over placebo in clinical samples through human laboratory trials, which are shorter and less expensive than RCTs. While many factors contribute to "go/no-go" decisions, the choice of human testing paradigms and the assessment of outcomes are crucial and affect the success rate of a compound's development (Plebani et al. 2012; Ray et al. 2010; Yardley and Ray 2017). The selection of models for human laboratory studies and the interpretation of their results, however, is often highly subjective (Egli 2018), potentially leading to biased "go/no-go" decisions and obstructing a stream of clinically effective medications. Aiming to lessen subjectivity and foster a data-driven approach in AUD medication development, this study evaluated whether medication effect sizes obtained using a common human laboratory paradigm (the alcohol cue exposure paradigm) are associated with effect sizes in RCTs for AUD.

Human laboratory models employ a variety of methods to examine core aspects of addiction in a controlled experimental setting. These models often expedite medication development in psychiatry by acting as a translational "bridge" from behavioral pharmacology to randomized clinical trials. The alcohol cue-reactivity paradigm, one of the most prevalent models, is frequently used (Meredith et al. 2023) in medication development for AUD. This protocol exposes participants to alcohol-related cues hypothesized to activate motivational mechanisms that drive alcohol use (Monti et al. 1987). Assessments of cue reactivity typically involve subjective measures like self-reported craving and physiological responses such as heart rate, used separately or in conjunction. During a standard in-vivo cue-reactivity test, participants provide baseline subjective and physiological data before the experimental tasks begin. They then interact with a "neutral" cue like water or juice, followed by an alcohol cue—usually their preferred alcoholic drink—and subsequently report their alcohol craving levels. The cue-reactivity paradigm not only shows high reproducibility (Carter and Tiffany 1999) but is also sensitive to medication effects. For example, alcohol cue-induced craving is blunted by FDA-approved medications for AUD, such as naltrexone (Miranda et al. 2014; Monti et al. 1999; O'Malley et al. 2002) and acamprosate (Hammarberg et al. 2009), as well as several other pharmacotherapies including, varenicline (Roberts et al. 2017), olanzapine (Hutchison et al. 2001), prazosin (Fox et al. 2012), and quetiapine (Ray et al. 2011).

Medication effects on human laboratory endpoints are hypothesized to predict drinking outcomes in RCTs; however, this hypothesis has not been routinely tested for alcohol cue-reactivity. A proof-of-concept study concerning a different laboratory model found that medication effects on subjective alcohol responses correlated with clinical trial outcomes. Specifically, our laboratory employed a novel meta-analytical approach to determine if medication effects on subjective alcohol response during alcohol administration in the human laboratory could predict RCT outcomes (Ray et al. 2021). We calculated medication effect sizes on stimulation, sedation, and craving during alcohol administration in the lab (51 studies involving 24 medications) and on abstinence and heavy drinking in RCTs (118 studies involving 17 medications). There was a significant relationship between alcohol-induced changes in stimulation, sedation, and craving and RCT outcomes. Medications that diminished stimulation and craving, and heightened sedation, were linked to improved clinical outcomes related to abstinence and heavy drinking in clinical trials. These findings are specific to alcohol administration phenotypes and should be extended to other methods frequently used in AUD medication development like the alcohol cue-reactivity paradigm. This line of work is especially timely as our laboratory recently showed that medication effect sizes on subjective response to alcohol are not highly correlated with medication effects on cue-induced alcohol craving (Ray et al. 2023).

The objective of the current study is to test whether medication effects in alcohol cue-reactivity studies are associated with medication effect sizes in RCTs for AUD. We conducted an extensive literature search for medications tested using the alcohol cue-reactivity paradigm and in RCTs. The descriptive statistics from each study were used to calculate medication effect sizes and the Williamson-York regression was used to test the relationship between medication effect sizes in alcohol cue-reactivity studies and medication effect sizes in RCTs. We hypothesized that medications that blunt alcohol cue-reactivity in the human laboratory, compared to placebo, will be associated with more favorable abstinence and heavy drinking endpoints in RCTs. We also used leave-one-out cross validation simulations to examine the predictive utility of cue-induced craving medication effect sizes on RCT endpoints at the level of each medication. Such information will provide the field with quantitative data to guide the use of the alcohol cue-reactivity paradigm as an early efficacy screening tool for AUD medications.

## Methods

### Literature review – alcohol cue-reactivity studies

The inclusion criteria for alcohol cue exposure studies were: (1) the use of a pharmacological agent (approved or in development for AUD) and a placebo or active control, (2) alcohol cue exposure in a controlled laboratory setting, including during brain imaging scans, (3) gathering self-reported craving in response to cues, and (4) articles published in peer-reviewed journals in English or translated to English. PubMed searches were performed on January 3, 2022, and the screening, coding, and analysis of studies continued until February 1, 2023.

The literature search strategy was informed by our laboratory’s previous meta-analysis. The previous meta-analysis tested the relationship between AUD medication effects on subjective response to alcohol and the outcomes of clinical randomized controlled trials (Ray et al. 2021). PubMed searches were conducted with assistance from UCLA librarians who have extensive expertise in systematic literature reviews. These searches utilized specific search and Medical Subject Headings (MeSH) terms related to alcohol response and craving, and were applied to each of the 40 medications previously identified. The terms used included "alcohol cue-exposure", "alcohol cue-reactivity", "ethanol craving", "alcohol craving", and MeSH terms like "Cues" and "Craving".

PubMed literature searches resulted in 358 unique studies. After screening the abstracts, 299 studies were excluded. A total of 59 studies underwent full-text review for eligibility, of which 23 were further excluded. Cue-reactivity articles were excluded at full-text review for the following reasons: ineligible trial design (*n* = 13), ineligible outcomes (*n* = 7). duplicate trial publication (*n* = 2), and ineligible comparator (*n* = 1). The final sample consisted of 36 cue-reactivity studies across 15 medications included in the current study. The main outcome measured in these studies was the craving experienced in response to alcohol cue exposure. The process and results of this literature search are documented in Preferred Reporting Items for Systematic Reviews and Meta-Analyses (PRISMA) flow charts provided in (Meredith et al. 2023).

The 36 cue-reactivity studies included a range of sample sizes, spanning from 11 participants (utilizing a crossover design) to 131 participants (employing a parallel design with two medication conditions). The median sample size among these studies was 39 participants, with 20 in the placebo condition and 22 in the medication condition. All studies provided data on participant enrollment based on biological sex with a mean of 71% male participants and 29% female participants. Approximately 75% of the studies (27 out of 36) had inclusion criteria that required individuals to meet DSM criteria for alcohol dependence or Alcohol Use Disorder (AUD). The remaining 25% of studies required participants to meet criteria for heavy drinking, although the specific definition of heavy drinking varied across trials. Please see (Meredith et al. 2023) for detailed study-level characteristics and cue-reactivity trial design features.

### Literature review – randomized clinical trials

Criteria for including randomized controlled trials (RCTs) were established as follows: (1) the study must be a randomized trial, (2) it must be either double-blinded or single-blinded, (3) it includes either a placebo or an active comparator, (4) the primary endpoint is on alcohol use, (5) it involves a minimum of four weeks of treatment with the medication, and (6) there is a follow-up period of at least 12 weeks post-randomization. We did not exclusively focus on 12-week clinical trials since some studies find medication effects at 4 weeks. A comprehensive search of the literature was previously carried out by our team up to July 2018 (Ray et al. 2021). The present research extends this search to include RCT literature published from January 2018 to April 2023. We refined the PubMed searches for the 17 AUD medications, using specific search and MeSH terms such as “randomized controlled trial”, “controlled clinical trial”, along with MeSH terms for alcohol-related interventions and therapies. A sample search pattern for the medication acamprosate has been provided for reference purposes: (((((randomized controlled trial[pt]) OR (controlled clinical trial[pt]) OR (randomized[tiab] OR randomised[tiab]) OR (placebo[tiab]) OR (drug therapy[sh]) OR (randomly[tiab]) OR (trial[tiab]) OR (groups[tiab]))) OR "Alcohol Deterrents/therapeutic use"[Mesh]) OR "Alcohol Drinking/drug therapy"[Mesh]) OR "Alcoholism/drug therapy"[Mesh] AND "alcohol" AND Acamprosate [tiab].

We obtained 207 unique studies from updated PubMed searches and excluded 165 of them during the abstract screening process. We then evaluated 42 studies for eligibility through full-text review, excluding 21 at this phase. RCT studies were excluded at full-text review for the following reasons: ineligible study design (*n* = 6), ineligible comparator (*n* = 3), and duplicate study (*n* = 12). We included 139 randomized controlled trial (RCT) studies pertaining to 19 medications in this study. The RCTs focused on several primary outcomes: Return to Any Drinking, Return to Heavy Drinking, Percent Days Abstinent, Percent Heavy Drinking Days, Drinks per Week, Drinks per Day, and Drinks per Drinking Day. A PRISMA flow chart detailing this literature search was reported in (Ray et al. 2021).

Across the 139 RCTs, the median sample size was 121 participants. The range of sample sizes spanned from a minimum of 10 participants to a maximum of 1383 participants in the multi-site COMBINE Study. The average age of participants was 44.7 years, with only 2 studies omitting age-related data. The median percentage of male participants in the entire dataset was 74%. When considering the distribution, 3 studies randomized between 0 to 40% male participants, 56 studies randomized between 40 to 60% male participants, and 80 studies randomized between 60 to 100% male participants.

At least two independent raters coded the endpoints and study-level descriptive information for all studies, including cue-reactivity and RCTs. In instances of coding discrepancies, raters convened to achieve consensus. We employed DigitizeIt software (Bormann 2012) as needed to extract descriptive statistics like means and standard errors from published figures (Rakap et al. 2016). Twenty cue-reactivity studies and two RCT studies required the use of Digitizeit software to extract the study means and standard errors. Additionally, we reached out to corresponding authors via email to request necessary data for effect size estimates when such data were not available in the publication. Data requests were sent to corresponding authors for 11 cue-reactivity studies and three RCT studies.

### Data analytic plan

#### Effect size estimation for cue-reactivity and RCT studies

We determined the unbiased Cohen’s *d* as the effect size for each cue-reactivity study, focusing solely on alcohol cue-induced craving. We defined Cohen’s *d* as the difference between the active medication group's mean and the control group's mean, divided by the pooled standard deviation ($\frac{{\overline{y} }_{medication}-{\overline{y} }_{control}}{\sqrt{\frac{\left({n}_{medication}-1\right){s}_{medication}^{2}+\left({n}_{control}-1\right){s}_{control}^{2}}{{n}_{medication}+{n}_{control}-2}}}$ where ${n}_{medication}$ and ${n}_{control}$ are the sample sizes of the treatment and control groups, ${\overline{y} }_{medication}$ and ${\overline{y} }_{control}$ are the sample means of the treatment and control groups, and ${s}_{medication}^{2}$ and ${s}_{control}^{2}$ are the sample variances of the treatment and control groups). The resultant effect size is usually called Hedges' *g* (Hedges 1981). However, Hedge’s *g* is widely known as a biased estimate of the population standardized group difference especially when the per-study sample size is small (Hedges 1981; Hedges and Olkin 2014). Hence, Hedges (1981) proposed an unbiased estimate, Cohen’s *d*, which was corrected by multiplying a correction factor to Hedge’s *g*, $d=1-\frac{3}{4\left({n}_{medication}+{n}_{control}\right)-9}$. Negative values of Cohen’s *d* suggested that the medication group experienced lower craving than the placebo group. For the RCTs, Cohen’s *d* was calculated for the Return to Any Drinking, Return to Heavy Drinking, Percent Heavy Drinking Days, Drinks per Week, Drinks per Day, and Drinks per Drinking Day endpoints and defined as the mean of the active medication group minus the mean of the control group divided by the pooled standard deviation. Cohen’s *d* was calculated for the Percent Days Abstinent endpoint and defined as the mean of the control group minus the mean of the active medication group divided by the pooled standard deviation. We calculated effect sizes associated with the active treatment period rather than follow-up periods for better consilience across studies since all studies reported data for treatment period but only a subset reported follow-up data. We then amalgamated effect sizes for certain heavy drinking outcomes (i.e., Return to Heavy Drinking, Percent Heavy Drinking Days, Drinks per Week, Drinks per Day, and Drinks per Drinking Day) into a single Heavy Drinking endpoint and did the same for Return to Any Drinking and Percent Days Abstinent to create an Abstinence endpoint. Medication effect sizes on the 7 individual outcomes are provided in supplemental materials. For both heavy drinking and abstinence endpoints used in statistical analyses, negative effect sizes indicated greater effectiveness of the treatment group over the control group.

We applied a random-effects meta-analysis using the *metafor* R package (Viechtbauer 2010) to calculate average effect sizes across trials for each medication, resulting in one average effect size per endpoint. The between-study heterogeneity is estimated using the widely used DerSimonian-Laird (DL) estimator (DerSimonian and Laird 1986). In cases where the number of studies is one, the between-study heterogeneity is fixed at 0. There were 15 medications assessed for craving in cue-reactivity studies and 19 for the Heavy Drinking and Abstinence endpoints in the RCT studies. Of these, 9 medication were tested in both cue-reactivity and RCT studies.

#### Cue-induced craving on RCT endpoints

We examined the correlation between medication effect sizes in cue-reactivity studies and RCTs using the Williamson-York bivariate weighted least squares estimation, which considers errors in both independent and dependent variables (Williamson 1968; York 1966; 1968; York et al. 2004). We employed two statistical models to compare effect sizes: one model regressed medication effect sizes on cue-induced craving and heavy drinking, and the other on cue-induced craving and abstinence.

We tested a one-tailed hypothesis where the alternative hypothesis was that the cue-induced craving slope would be greater than zero (${H}_{a}:\beta >0$: vs. ${H}_{0}:\beta =0$). We employed the Wald test by comparing the observed Z-score to the one-sided critical value of 1.64. To account for multiple hypothesis testing, we adjusted the significance level, dividing the alpha level of 0.05 by the number of regression models, which set the corrected alpha level at 0.025 for each test. We carried out sensitivity analyses to adjust for publication bias, employing the *p*-uniform method (van Aert et al. 2016) to derive corrected estimates of overall effect sizes and then performed regression analysis. For this analysis, we utilized the *puniform* R package (Van Aert 2017). The results with publication bias correction should be interpreted with caution as the correction is better suited for fixed-effects meta-analysis and it only uses the information of significant effect sizes.

#### Predictive accuracy of cue-induced craving on RCT endpoints

We used leave-one-out validation to illustrate the predictive accuracy of cue-craving effect sizes on the Abstinence and Heavy Drinking RCT endpoints at the level of each study medication. More specifically, the Williamson-York regression models were trained on a dataset with a single medication removed (the target medication). The regression models were then used to predict the RCT effect size of the target medication based on its cue-craving effect size. For each target medication, we generated a predictive distribution for the RCT effect size with 10^4 predicted values based on the Williamson-York regression coefficients, the corresponding cue-craving effect size, and cue-craving effect size’s standard error.

## Results

### Cue-induced alcohol craving and heavy drinking

**Fig. 1:**
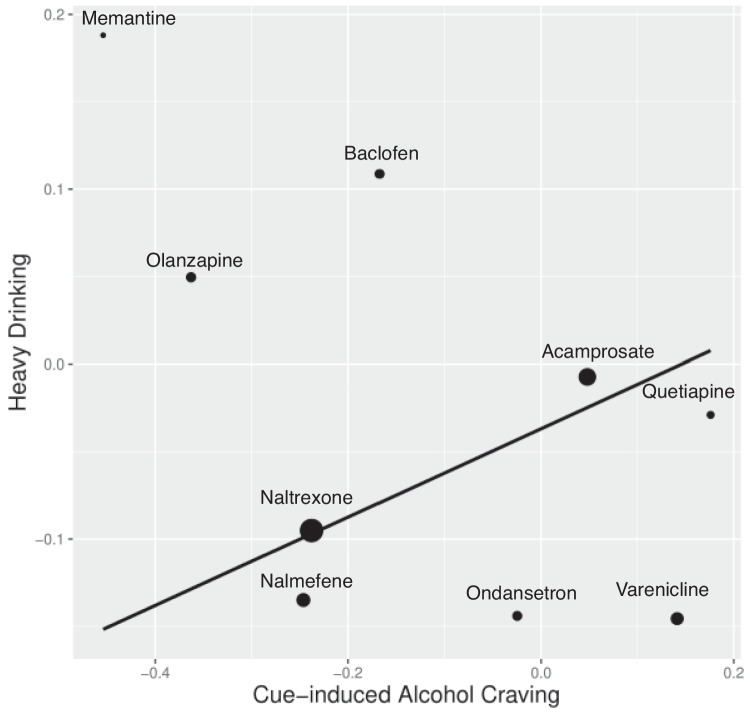
Medications, each represented and labeled as a dot on the regression line, show a linear relationship between their effect sizes on RCT heavy drinking and their effect sizes on cue-induced craving. Smaller dots indicate greater error variance, and larger dots indicate less error variance around each estimate. Negative medication effect sizes indicate a more favorable medication effect

We applied the Williamson-York regression to assess the relationship between effect sizes for heavy drinking in RCTs and those for cue-induced alcohol craving in cue-reactivity studies. The aim was to determine if there was a correlation between the effect sizes for heavy drinking and those for cue-induced craving across the two types of studies. In Fig. 1, each medication is represented by a dot, where larger dots denote smaller sampling errors and thus carry more weight. The x-axis represents the effect sizes for heavy drinking from RCTs, while the y-axis reflects the effect sizes for cue-induced alcohol craving. Nine AUD medications are depicted in Fig. 1. Previous reports of effect size estimations for cue-induced craving can be found in (Meredith et al. 2023) and (Ray et al. 2023). We present the effect size estimations for heavy drinking from RCTs, derived from the updated literature search, in Table 1.

**Table 1:** Estimated effect sizes and standard errors for each medication for the RCT heavy drinking and cue-craving outcome

Medication	Number of Heavy Drinking Effect Sizes	Cohen’s d for Heavy Drinking	Standard Error for Heavy Drinking	Number of Cue-craving Effect Sizes	Cohen’s d for Cue-craving	Standard Error for Cue-craving
Acamprosate	112	−0.007	0.020	4	0.048	0.140
Aripiprazole	4	−0.099	0.098			
Baclofen	28	0.109	0.100	10	−0.167	0.134
Carbamazepine	4	0.105	0.235			
Gabapentin	24	−0.495	0.113			
Levetiracetam	12	0.021	0.059			
Memantine	4	0.188	0.193	2	−0.454	0.164
Nalmefene	28	−0.135	0.030	3	−0.246	0.193
Naltrexone	176	−0.095	0.020	12	−0.238	0.077
Olanzapine	8	0.050	0.082	3	−0.363	0.187
Ondansetron	12	−0.144	0.066	1	−0.025	0.292
Quetiapine	20	−0.029	0.045	1	0.176	0.541
Rimonabant	4	−0.066	0.065			
Ritanserin	12	−0.088	0.089			
Sertraline	4	0.000	0.111			
Topiramate	40	−0.263	0.056			
Valproate	12	−0.334	0.114			
Varenicline	16	−0.146	0.052	4	0.141	0.148
Zonisamide	8	−0.221	0.147			

Negative medication effect sizes indicate a more favorable medication effect

The estimated slope is $\widehat{\beta }$ = 0.253 (SE = 0.189, *p* = 0.090). Since this does not meet the threshold of the corrected α level, the slope is not statistically significant. While the positive slope is in the expected direction, we cannot assert a positive linear relationship between the effect sizes for heavy drinking in RCT studies and those in cue-reactivity studies. The conclusion remained largely unchanged after publication bias correction ($\widehat{\beta }$= 0.823, SE = 0.638, *p* = 0.098).

We also performed Monte Carlo power analysis. Based on the estimated slope and the effect size of cue-induced alcohol craving, we simulated 9 medication effect sizes for heavy drinking. Based on these simulated effect sizes, observed cue-induced alcohol craving effect sizes and their respective standard errors, we conducted Williamson-York regression analysis. This procedure was replicated 10,000 times, with statistical power computed by counting the number of observed Z scores larger than the one-sided critical value of 1.64. The Monte Carlo power for heavy drinking was estimated to be 0.9776.

### Cue-induced alcohol craving and abstinence

**Fig. 2:**
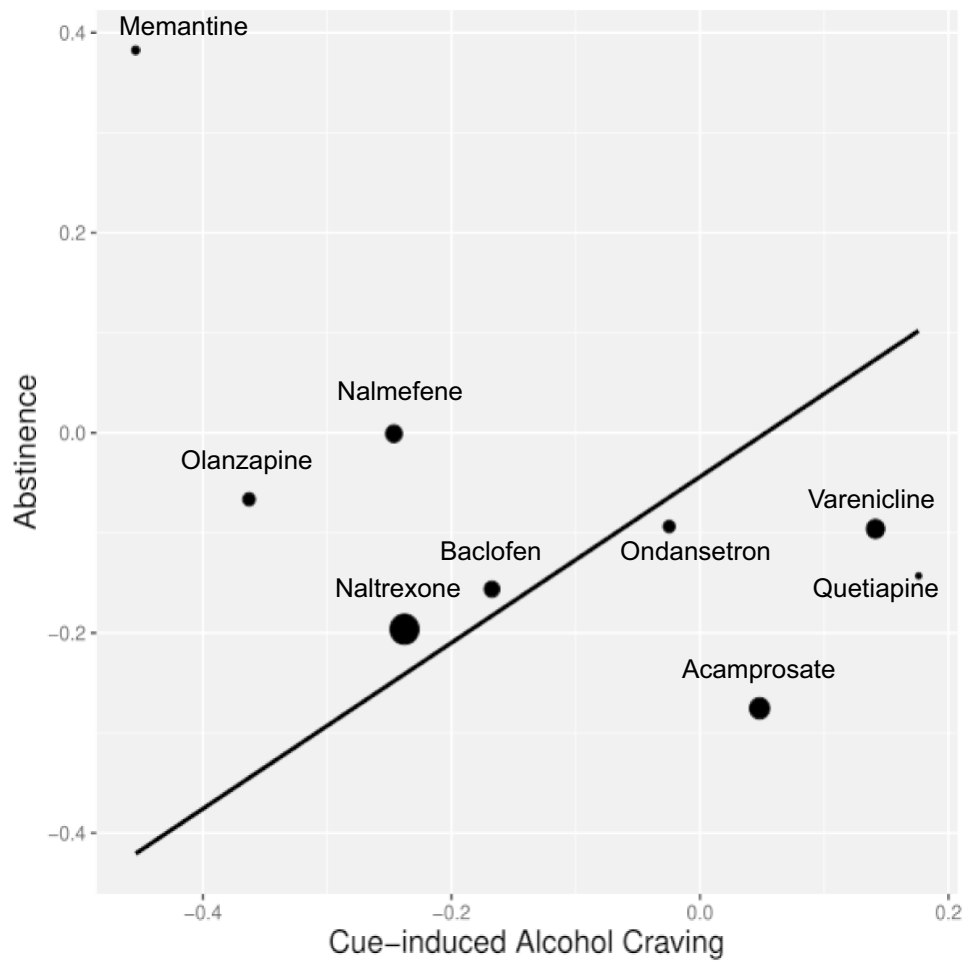
Medications, each represented and labeled as a dot on the regression line, show a linear relationship between their effect sizes on RCT abstinence and their effect sizes on cue-induced craving. Smaller dots indicate greater error variance, and larger dots indicate less error variance around each estimate. Negative medication effect sizes indicate a more favorable medication effect

We tested the linear relationship between the medication effect sizes of abstinence within RCT studies and those of cue-induced alcohol craving within cue-reactivity studies. Data was available for 9 medications. The linear slope is not significant ($\widehat{\beta }$ = 0.829, SE = 0.747, *p* = 0.133; see Fig. 2). Hence, we fail to conclude that medications that decreased craving in cue-reactivity studies increased abstinence in the RCT studies. The conclusion remained the same with publication bias correction ($\widehat{\beta }$ = -3.385, SE = 6.671, *p* = 0.694). Effect size estimations from the updated RCT literature search for abstinence are presented in Table 2.

**Table 2:** Estimated effect sizes and standard errors for each medication for the RCT abstinence outcome

Medication	Number of Effect Sizes	Cohen’s *d*	Standard Error
Acamprosate	56	−0.275	0.053
Aripiprazole	2	0.261	0.155
Baclofen	14	−0.156	0.147
Carbamazepine	2	−0.211	0.289
Gabapentin	12	−0.180	0.090
Levetiracetam	6	0.016	0.080
Memantine	2	0.382	0.388
Nalmefene	14	−0.001	0.036
Naltrexone	88	−0.196	0.049
Olanzapine	4	−0.066	0.189
Ondansetron	6	−0.094	0.093
Quetiapine	10	−0.143	0.104
Rimonabant	2	−0.114	0.095
Ritanserin	6	−0.009	0.134
Sertraline	2	−0.049	0.175
Topiramate	20	−0.183	0.065
Valproate	6	−0.004	0.139
Varenicline	8	−0.096	0.079
Zonisamide	4	−0.220	0.214

Negative medication effect sizes indicate a more favorable medication effect

For abstinence, we performed Monte Carlo power analysis similar to heavy drinking. The Monte Carlo power for abstinence was estimated to be 0.9997.

### Predictive accuracy of cue-reactivity effect sizes on heavy drinking and abstinence

**Fig. 3:**
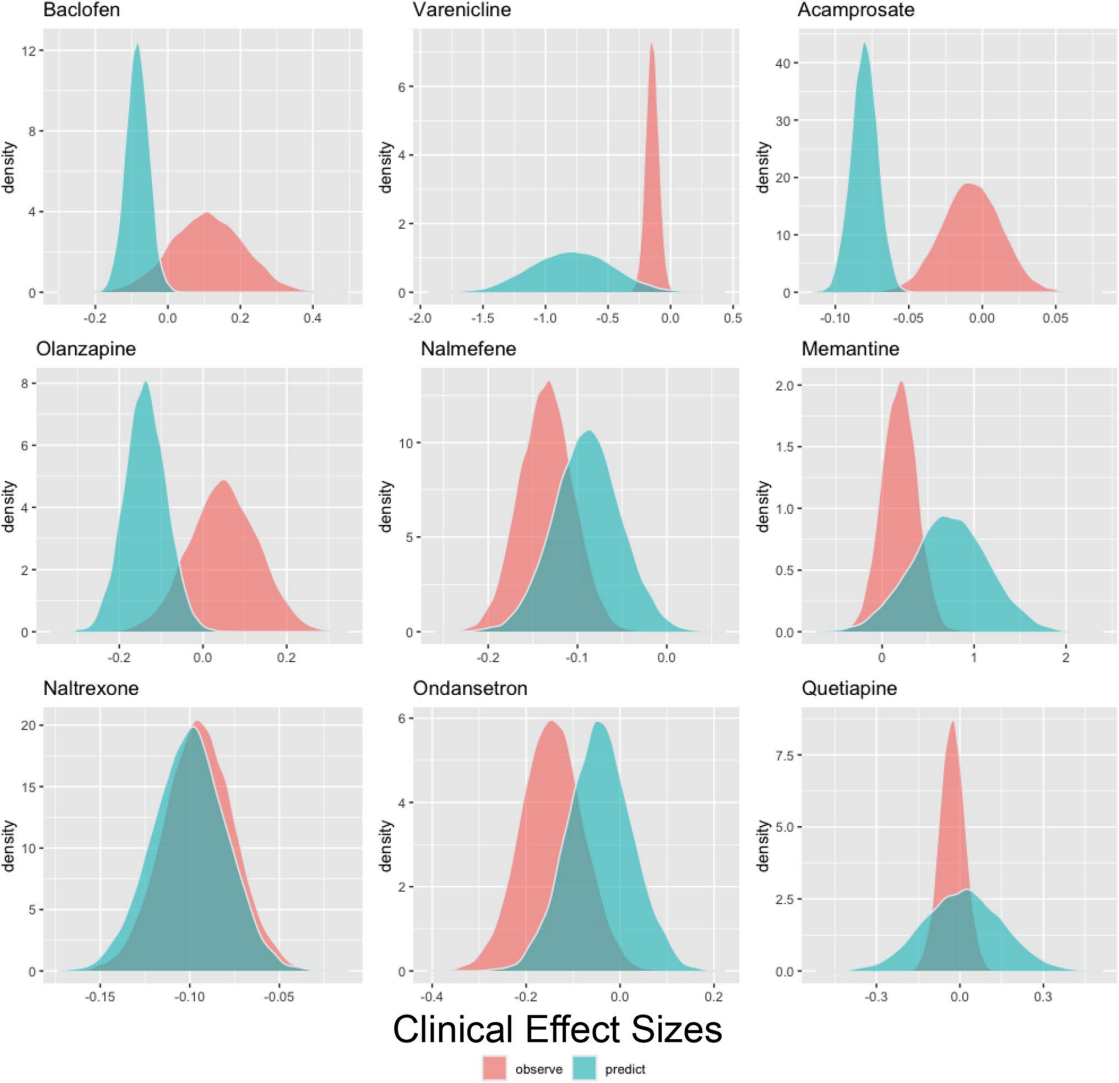
Predicted distributions of effect sizes for heavy drinking were created for each medication by applying a leave-one-out simulation approach across cue-induced craving effect sizes. In cases where more than cue-induced craving was present for a medication, these predicted distributions were combined. Negative medication effect sizes indicate a more favorable medication effect

**Fig. 4:**
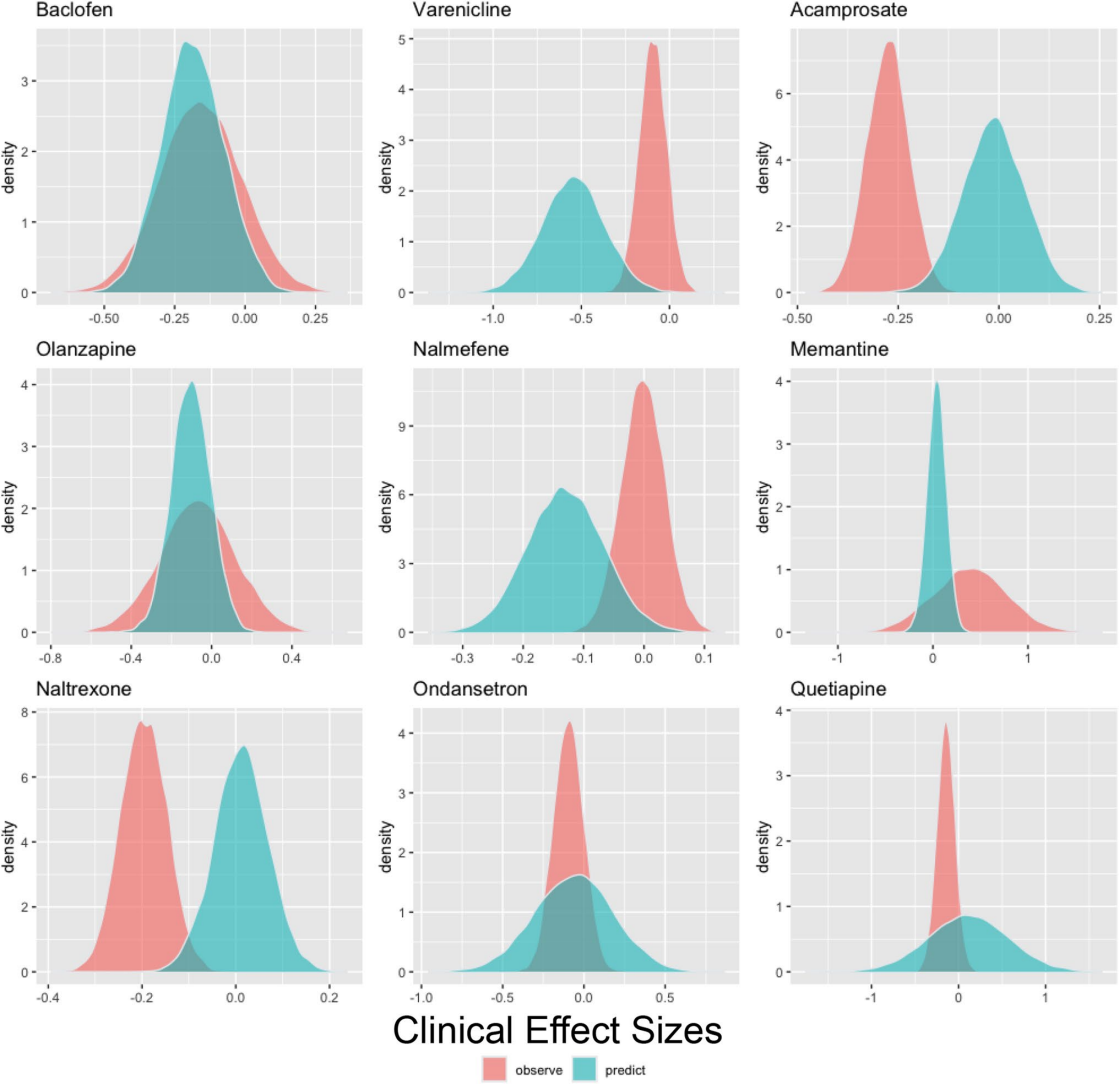
Predicted distributions of effect sizes for abstinence were created for each medication by applying a leave-one-out simulation approach across cue-induced craving effect sizes. In cases where more than cue-induced craving was present for a medication, these predicted distributions were combined. Negative medication effect sizes indicate a more favorable medication effect

We tested the predictive **accuracy** of medication effects on cue-induced craving on medication effect sizes on RCT abstinence and heavy drinking effect sizes. The predictive distribution of medication effects on cue-induced craving generally covered the estimated sampling distribution for RCT heavy drinking effect sizes, except for Baclofen and Acamprosate (see Fig. 3 and Table 3). The predictive distribution of medication effects on cue-induced craving generally also covered the estimated sampling distribution for RCT abstinence effect sizes, except for Acamprosate and Naltrexone (see Fig. 4 and Table 4).

**Table 3:** Predicted clinical effect size for heavy drinking based on each medications’ cue-craving effect size using a leave-one-out cross validation simulation method on the Williamson-York regression models

Medication	Number of Effect Sizes	Observed Effect Size	Predicted Effect Size	Standard Error of the Predicted Effect Size
Acamprosate	112	−0.007	−0.080	0.009
Baclofen	28	0.109	−0.085	0.031
Memantine	4	0.188	0.733	−0.419
Nalmefene	28	−0.135	−0.089	0.038
Naltrexone	176	−0.095	−0.100	0.020
Olanzapine	8	0.050	−0.138	0.049
Ondansetron	12	−0.144	−0.042	0.069
Quetiapine	20	−0.029	0.012	0.142
Varenicline	16	−0.146	−0.789	−0.328

Negative medication effect sizes indicate a more favorable medication effect

**Table 4:** Predicted clinical effect size for abstinence based on each medications’ cue-craving effect size using a leave-one-out cross validation simulation method on the Williamson-York regression models

Medication	Number of Effect Sizes	Observed Effect Size	Predicted Effect Size	Standard Error of the Predicted Effect Size
Acamprosate	56	−0.275	−0.012	0.076
Baclofen	14	−0.156	−0.185	0.111
Memantine	2	0.382	0.040	−0.099
Nalmefene	14	−0.001	−0.129	−0.063
Naltrexone	88	−0.196	0.012	−0.059
Olanzapine	4	−0.066	−0.103	0.097
Ondansetron	6	−0.094	−0.061	0.246
Quetiapine	10	−0.143	0.112	0.459
Varenicline	8	−0.096	−0.544	−0.178

Negative medication effect sizes indicate a more favorable medication effect

## Discussion

Medication development for AUD is critical to improving healthcare for individuals affected by this highly prevalent disorder. Identifying human laboratory paradigms that can be leveraged to screen medications efficiently while able to detect an early efficacy signal have the potential to streamline the medication development process. The alcohol cue-reactivity paradigm is one of the most widely used behavioral pharmacology paradigms. While the cue-reactivity paradigm is sensitive to medication effects, there is only qualitative support for cue-reactivity findings predicting clinical outcomes. The purpose of this study was to reduce subjectivity in using this paradigm as an early efficacy signal by assessing the quantitative evidence for the predictive utility of the cue-reactivity paradigm on clinical trial outcomes. To do this, we leveraged our laboratory’s extensive systematic literature reviews and meta-analyses of medications tested in cue-reactivity studies (Meredith et al. 2023; Ray et al. 2023) and randomized clinical trials (Ray et al. 2021) to investigate the relationship between medication effects on cue-reactivity and medication effects on RCTs endpoints, namely heavy drinking and abstinence. Results showed that there was no significant association between cue-induced craving and RCT heavy drinking, such that medications that reduced cue-induced craving during the cue-exposure paradigm were not more likely decrease heavy drinking in RCTs. The direction of the slope, showing a positive association, was in support of our hypothesis. There was also no significant association between cue-induced craving and RCT abstinence, such that medications that reduced cue-induced craving during the cue-exposure paradigm were not more likely to increase abstinence in RCTs.

These results are unexpected given the widespread use of cue-reactivity paradigms in medication development for AUD and highlight the need for other paradigms/outcomes in human laboratory testing. Some studies suggest that reduction in craving is a pivotal mechanism of action in effective treatment (Kosten 1992; Weiss et al. 2003) for addiction. While the current study suggests low predictive utility of cue-induced alcohol craving alone in predicting clinical outcomes in RCTs, removal or avoidance of alcohol-related cues remains an important treatment target especially for people in early recovery. Thus, developing behavioral and pharmacological interventions that directly target craving mechanisms are still warranted (Lopez et al. 2022). It is important to note that the cue-reactivity studies included in the current study had small sample sizes and did not routinely test for baseline reactivity and alcohol craving which have been shown to influence medication effects (Meredith et al. 2023). Additionally, the current study included medications with varying mechanisms of action and perhaps certain medications may be reducing drinking through means other than reducing cue-induced craving.

We also conducted leave-one-out cross validation analyses to assess the predictive utility of the cue-craving effect sizes on RCT heavy drinking and abstinence endpoints at the level of each study medication. It is important to note that the uncertainty in effect sizes is relatively large, primarily due to the precision of cue-craving effect size measurements and the moderate correlations between cue-craving and RCT effect sizes. Although there was generally a strong agreement between predicted and observed effect sizes, there were a few noteworthy exceptions. Specifically, nalmefene and ondansetron were found to have a more substantial positive clinical impact on RCT heavy drinking rates than anticipated, while baclofen, varenicline, and acamprosate exhibited a notably more negative clinical effect than predicted. Interestingly, for the RCT abstinence endpoint, naltrexone and acamprosate had a more positive clinical impact than expected while varenicline and nalmefene had a more negative clinical impact than expected. It may be the case that for antagonist medications like nalmefene, reducing heavy drinking may be the primary desired outcome (Bahji et al. 2022), while for agonist medications like acamprosate, complete abstinence may be a more favorable goal. In addition to refining behavioral pharmacology testing by selecting laboratory endpoints aligned with the mechanism of action of a given medication (i.e., agonist or antagonist), similar refinement should be considered when choosing clinical outcomes. In summary, the simulation analyses provide medication-specific findings and directly assess the predictive utility of cue-craving effect sizes on relevant RCT endpoints. Notably, participants in clinical trials vary in their treatment goals and as such are not uniformly motivated to pursue abstinence from alcohol during the trial (Bujarski et al. 2013).

This study includes a comprehensive examination of AUD medications tested using the cue-reactivity paradigm in the human laboratory and in RCTs. Strengths of this study include the novel methods that allow for integration of findings across levels of analyses by allowing for independent error terms in both dependent and independent variables, as well as large number of RCT effect sizes. Limitations of this study include the fact that a few studies could not be included in the analyses due to limited medication effect sizes available (i.e., physiological endpoints from cue-reactivity studies) and/or for lack of proper data reporting (Meredith et al. 2023). The literature searches for this project were limited to published studies. Nevertheless, the directionality of the findings suggest that a wide host of studies would be needed to support the initial hypothesis that medications that reduce alcohol cue-reactivity in the laboratory are more likely to reduce heavy drinking and to promote abstinence in clinical trials. Another important limitation is that the analyses cannot differentiate between medications designed to counteract the rewarding effects of alcohol, such as naltrexone and nalmefene, and medications that aim to promote abstinence by restoring balance in brain systems disrupted during abstinence, such as acamprosate. The present study, while harnessing effect sizes across 40 years of research, lacks the necessary statistical power to make such distinctions. Nevertheless, it may be the case that the behavioral pharmacology experiments involving alcohol cue-induced craving in a laboratory setting might be more suitable for evaluating antagonist medications We speculate that perhaps antagonist medications were developed first and as such, the successful models for developing them have been adopted as a constant in the field. As novel compounds are developed, the screening models for human laboratory studies should also encompass a wider range of possible mechanisms beyond cue-induced alcohol craving.

On balance, this study does not support the widely held assumption that alcohol cue-reactivity in the human laboratory represents an early efficacy marker for AUD medications, such that there was no significant association between medication effect sizes on cue-reactivity in the laboratory and drinking outcomes in RCTs. These findings suggest that a wider host of early efficacy indicators be considered for early Phase II testing of novel compounds. This is critical to prevent false-negatives (i.e., a no-go decision on a compound that may actually be effective in clinical settings) based solely on alcohol cue-reactivity as an early efficacy indicator. To that end, the inclusion of drinking outcomes in addition to alcohol cue-reactivity (e.g., (Miranda et al. 2020)) represents a possible solution to further validate the degree to which medication effects on alcohol cue-reactivity “tracks” with changes in alcohol use itself. In closing, this study challenges the assumption that alcohol cue-reactivity alone can be used as an early efficacy indicator for AUD pharmacotherapy development.

### Supplementary Information

Below is the link to the electronic supplementary material.

Supplementary file1 (XLSX 48 KB)
